# From tetraploid to diploid, a pangenomic approach to identify genes lost during synthetic diploidization of *Eragrostis curvula*

**DOI:** 10.3389/fpls.2023.1133986

**Published:** 2023-03-13

**Authors:** Jose Carballo, Andrés Martin Bellido, Juan Pablo Selva, Diego Zappacosta, Cristian Andres Gallo, Emidio Albertini, Mario Caccamo, Viviana Echenique

**Affiliations:** ^1^ Centro de Recursos Naturales Renovables de la Zona Semiárida (CERZOS), Universidad Nacional del Sur-Consejo Nacional de Investigaciones Científicas y Técnicas (CONICET), Bahía Blanca, Argentina; ^2^ Departamento de Biología, Bioquímica y Farmacia, Universidad Nacional del Sur (UNS), Bahía Blanca, Argentina; ^3^ Departamento de Agronomía, Universidad Nacional del Sur (UNS), Bahía Blanca, Argentina; ^4^ Dipartimento di Scienze Agrarie, Alimentari e Ambientali, Università degli Studi di Perugia, Perugia, Italy; ^5^ NIAB, Cambridge, United Kingdom

**Keywords:** diploidization, *Eragrostis curvula*, plant reproduction, ploidy, apomixis

## Abstract

**Introduction:**

In *Eragrostis curvula*, commonly known as weeping lovegrass, a synthetic diploidization event of the facultative apomictic tetraploid Tanganyika INTA cv. originated from the sexual diploid Victoria cv. Apomixis is an asexual reproduction by seeds in which the progeny is genetically identical to the maternal plant.

**Methods:**

To assess the genomic changes related to ploidy and to the reproductive mode occurring during diploidization, a mapping approach was followed to obtain the first *E. curvula* pangenome assembly. In this way, gDNA of Tanganyika INTA was extracted and sequenced in 2x250 Illumina pair-end reads and mapped against the Victoria genome assembly. The unmapped reads were used for variant calling, while the mapped reads were assembled using Masurca software.

**Results:**

The length of the assembly was 28,982,419 bp distributed in 18,032 contigs, and the variable genes annotated in these contigs rendered 3,952 gene models. Functional annotation of the genes showed that the reproductive pathway was differentially enriched. PCR amplification in gDNA and cDNA of Tanganyika INTA and Victoria was conducted to validate the presence/absence variation in five genes related to reproduction and ploidy. The polyploid nature of the Tanganyika INTA genome was also evaluated through the variant calling analysis showing the single nucleotide polymorphism (SNP) coverage and allele frequency distribution with a segmental allotetraploid pairing behavior.

**Discussion:**

The results presented here suggest that the genes were lost in Tanganyika INTA during the diploidization process that was conducted to suppress the apomictic pathway, affecting severely the fertility of Victoria cv.

## 1 Introduction

In the last few years, the genome assembly paradigm, by which one reference genome was considered enough to represent the whole diversity of a given species, is shifting to a new method, called pangenome, to represent the genetic diversity within a single species. The requirement of researchers to cover more diversity and the decreasing cost of genome sequencing were the two key components for the emergence of the pangenome concept (https://www.genome.gov/, accessed 12 November 2022). A pangenome assembly is defined by the core genome, which is shared by all the individuals analyzed, and the variable or dispensable genome, which is only present in some of the individuals (Tettelin et al., 2005).

The methodological procedure to assemble a pangenome is not standardized yet. However, the most used pipeline so far is the *Iterative* mapping approach (Golicz et al., 2016b; Gao et al., 2019; Ruperao et al., 2021). Through this process, DNA sequencing reads from different genotypes are mapped against a reference genome assembly. The mapped reads are used to call variants, while the unmapped ones are assembled in new genomic fragments in order to annotate genes not present in the reference genome. After that, the newly assembled sequences are merged to the reference genome and the process starts again by mapping another sample (Golicz et al., 2016a). Once all the individuals are processed, the obtained pangenome contains the reference genome plus the newly assembled sequences.

In plant biology, the pangenome model is especially important, since the majority of the reference genomes sequenced were selected to represent an entire species in one genome and to study individual genomes as models to translate the results to orphan species. For example, *Arabidopsis thaliana* and rice (*Oryza sativa*) are the models used to study dicots and monocots species, respectively (Rensink and Buell, 2004). These species are also used as proofs of concept and for functional validation. In wheat, the first near complete genome assembly was obtained from the Chinese spring cultivar, which is distantly related to modern cultivars (Zimin et al., 2017). Therefore, a wheat pangenome was later sequenced including modern varieties, finding 12,150 variables genes that were absent in Chinese Spring and 225,064 non-synonymous single nucleotide polymorphisms (SNPs) (Montenegro et al., 2017). Other modern wheat varieties were sequenced by the 10+ Genome Project, with the aim of characterizing the wheat pangenome and annotating high-confidence gene models (Walkowiak et al., 2020). Recent publications indicate that in economically important crops like barley and rice, the pangenome approach is displacing the genome reference one (Jayakodi et al., 2020; Qin et al., 2021). Pangenomic assemblies of major crops successfully increase the number of genes related to agronomically important traits. In soybean (*Glycine max*), the pangenome assembly of seven individuals allowed to discover gene presence/absence variations (PAVs) and mutations related to biotic resistance, seed composition, flowering and maturity time, organ size, and final biomass not previously recorded (Li et al., 2014). Another interesting example comes from the *Brassica oleracea* pangenome, obtained from nine varieties that allowed to discover 11,484 genes not annotated in the reference genome, including those associated with disease resistance, flowering time, glucosinolate metabolism, and vitamin biosynthesis (Golicz et al., 2016b). Finally, in cotton, a pangenome assembled with genomic data from 1,961 individuals evidenced the presence of several genes of agronomic relevance that were lost during domestication (Li et al., 2021a).

Hundreds of plants were sequenced since the genome assembly of *Arabidopsis thaliana* two decades ago, showing the complexity and diversity of plant genomes (Arabidopsis Genome Initiative, 2000). From many evolutionary studies, it is already known that a paleoploidization event occurred 160 million of years ago affecting all the flowering plants (Callaway, 2013). After that, the majority of the species recovered the diploid level by two processes, namely, cytological diploidization and gene diploidization. Cytological diploidization was the result of fission, fusion, and other large-scale chromosomal rearrangements, while in gene diploidization, many duplicated genes were evolutionary lost, and only their subset was retained (Li et al., 2021b). Both processes eventually produced diploid-like chromosome pairing during meiosis, directing the formation of a diploid genotype. Anthers culture is another mechanism of synthetic diploidization in which the haploid pollen is regenerated into a plant. When the maternal plant is diploid, a duplication is required since the pollen is haploid, while in tetraploids, viable plants can be obtained from diploid pollen (Cho and Zapata, 1988; Christensen et al., 1997; Chen et al., 2016).

In *Eragrostis curvula*, a perennial grass commonly known as weeping lovegrass, a diploidization event was obtained synthetically from a facultative apomictic tetraploid plant. Briefly, young inflorescences from Tanganyika INTA cv. (2n=4x=40) were *in vitro* cultured passing through a callus stage, and after 4 months, the regenerated plants were transferred to pots with soil. One of the plants, the 23 *R_0_*, was shown to have 20 chromosomes instead of the normal 40 of the maternal plant, and it was fully sexual (Cardone et al., 2006). This plant was registered as Victoria cv. The hypothesis of this diploidization event was that a reduced microspore of Tanganyika INTA gave rise to Victoria. Since Tanganyika INTA is facultative apomictic, the probability that Victoria has arisen from a reduced megasporocyte is very low because the percentage of sexual embryos in this genotype is normally below 5% (Rodrigo et al., 2017).

Apomixis is an asexual reproduction by seeds in which the offspring is genetically identical to the maternal plant. Apomictic plants of *E. curvula* have their own type of embryo sac development, called *Eragrostis* type (Crane, 2001), instead of the *Polygonum* type found in sexual plants of the species. In this type of diplosporous apomixis, meiosis is completely circumvented, the embryo arises without fertilization, and one sperm cell of the pollen fertilizes the central cell to form the endosperm. These three processes are defined as apomeiosis, parthenogenesis, and pseudogamy, respectively. Even though many studies of the group at transcriptomic, genomic, and epigenomic levels were taken and several advances were made to elucidate the apomixis mechanisms in *E. curvula*, the key molecular components regulating this pathway have not yet been resolved (Cervigni et al., 2008a; Cervigni et al., 2008b; Garbus et al., 2017; Selva et al., 2017; Carballo et al., 2019; Garbus et al., 2019; Zappacosta et al., 2019; Selva et al., 2020; Carballo et al., 2021a; Pasten et al., 2022).

The assembled genome of the sexual diploid Victoria is already available at chromosome level (Carballo et al., 2019). Our aim is to use this genome as reference to follow a pangenomic approach to assess the genomic changes that occurred during the recent diploidization of the tetraploide apomictic Tanganyika INTA that produced the genotype Victoria, a sexual diploid genotype. Thus, the results presented here suggest that the genes lost in Tanganyika INTA produced the modification of the reproductive mode from apomictic to sexual, and as a consequence, the options in terms of reproductive mechanism were reduced, affecting seriously the fertility of Victoria cv.

## 2 Materials and methods

### 2.1 Plant material and sequencing

DNA was obtained from a Tanganyika INTA plant growing in the greenhouse at CERZOS, CCT-CONICET Bahía Blanca, Argentina. The cetyltrimethyl ammonium bromide (CTAB)-based method, previously tested in *E. curvula*, was used for DNA extraction starting from 80 mg of fresh leaf tissue (Meier et al., 2011). After library preparation, the DNA was sequenced in pair-end 2x250 bp in a HiSeq 1500 Illumina machine rendering ~118 Gbp. These data were deposited in NCBI database under the bioproject PRJNA914706.

### 2.2 RNA extraction

For RNA extraction, the Promega SV Total RNA Isolation System^©^ was used. In brief, up to 60 mg of tissue was processed, and the RNA was separated, precipitated, and bounded using the spin baskets containing a silica membrane. Later, RNA was treated with a RNase-Free DNase I and eluted with nuclease-free water. The quality and quantity of the RNA was assessed using the DeNovix DS-11 Spectrophotometer and an Agarose Gel protocol (Aranda et al., 2012). Afterwards, first-strand cDNA was performed using the NEB M-MuVL Reverse Transcriptase^©^. Finally, PCRs were conducted through the PB-L TAQ Pegasus^©^ protocol, and amplicons were analyzed by electrophoresis in 1% (m/v) agarose gel.

### 2.3 Flow cytometry

Flow cytometry was used for genome size determinations of plants of cvs. Victoria and Tanganyika INTA using the service of the Instituto de Genética, INTA Castelar (Castelar, Buenos Aires, Argentina) according to the protocol of Galbraith et al. (1983). Fresh leaves from each genotype were stained with propidium iodide, and the samples were examined by a Partec PA II flow cytometer. For genome size estimation in base pairs, the fluorescence of each cultivar in relative fluorescence units (RFUs) was contrasted against the *Secale cereale* genome (7.917 Mb), since the flow cytometry peak of this species did not overlap with that expected for *E. curvula* (Doležel et al., 1998).

### 2.4 Pangenome assembly

**Figure 1:**
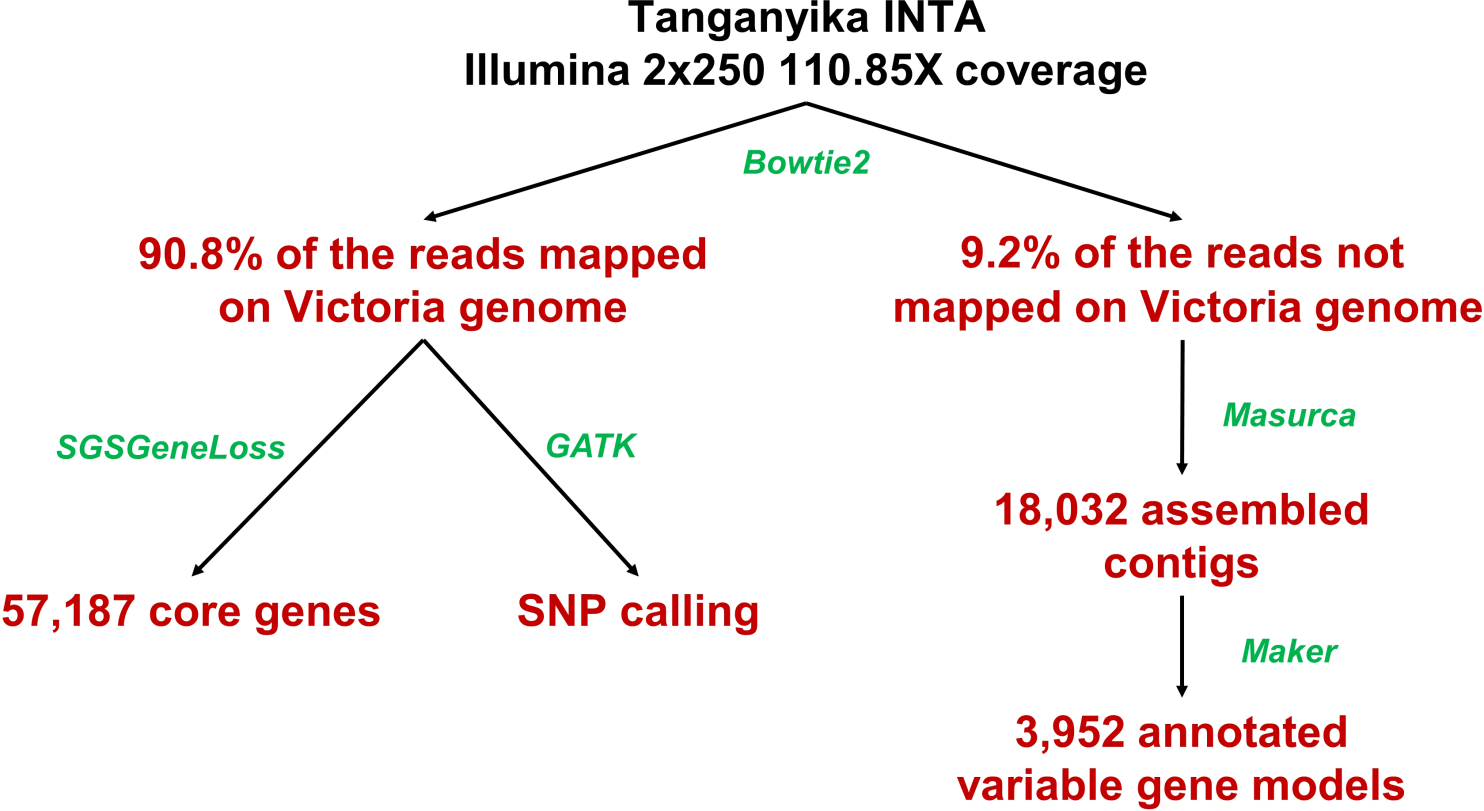
Pipeline used to assemble the *Eragrostis curvula* pangenome. Programs used are highlighted in green and the result in red.

For the pangenome assembly, the iterative mapping method was used (**Figure 1**) (Golicz et al., 2016b; Zhao et al., 2020; Ruperao et al., 2021). Tanganyika INTA reads were mapped against the Victoria reference genome (Carballo et al., 2019; NCBI bioproject PRJNA508722) using Bowtie2 software (parameters -I 0 -X 1,000) (Langmead and Salzberg, 2012). The Victoria cultivar was obtained from *in vitro* culture of Tanganyika INTA young inflorescences (Echenique et al., 1996), and the genome sequencing was made from DNA of the original R1 plant. The unmapped reads were extracted from the Bowtie2 output and assembled with Masurca V3.2.8 software (Zimin et al., 2013). The assembled contigs were aligned against the NCBI Viridiplantae plastid database in order to discard possible contaminations using the blast alignment tool (Altschul et al., 1990). Then, to remove redundancy, the assembly was self-compared with nucmer aligner filtering sequences >90% identity and >90% coverage (Marçais et al., 2018). The remaining contigs with more than 1,000 bp were included in the assembly.

### 2.5 Pangenome annotation

Repetitive elements included in the newly assembled contigs were annotated and classified using the EDTA software (Ou et al., 2019). For gene annotation, the software maker (Holt and Yandell, 2011) was implemented using RNA-seq data from *E. curvula* and *E. tef* and protein sequences from related species such as *Eragrostis nindensis*, *Setaria italica*, *Panicum halli*, and *Oropetium thomaeum* (Zhang et al., 2012; Lovell et al., 2018; VanBuren et al., 2018; Garbus et al., 2019; Pardo et al., 2020; Selva et al., 2020; VanBuren et al., 2020). The gene models obtained through this process were functionally annotated using as reference the UniProt database (Ruch et al., 2021). For this task, Blastp software was used with an e-value cutoff of 0.001 (Altschul et al., 1990). Gene Ontology (GO) was based on Interproscan 5 annotation (Jones et al., 2014), and the GO enrichment analysis of the variable genes was performed using the R package clusterProfiler (Yu et al., 2012) with a p-value adjusted cutoff of 0.05.

### 2.6 Gene presence/absence variations

Genomic sequence data from Tanganyika INTA and transcriptomic data from *E. curvula* cv. Don Walter (Selva et al., 2020) were mapped against the pangenome using Bowtie2 (Langmead and Salzberg, 2012). Gene presence/absence variations were assessed with the SGSGeneLoss software (Golicz et al., 2015) in order to perform phylogenetic analysis. After that, a matrix was constructed to perform phylogenetic analysis using R software (Team, R. Core, 2018).

### 2.7 Variant calling

SNP calling was performed following the GATK best practice protocol (McKenna et al., 2010). Briefly, Tanganyika INTA reads were converted to bam, and the adapters were marked with Picard tools (https://broadinstitute.github.io/picard/). Then, the resulted bam was converted back to fastq and mapped against the genome with BWA-MEM software. Finally, SNP calling and filtering was performed based on mapping quality and coverage.

### 2.8 Gene presence/absence validations

Genes present in Tanganyika INTA and absent in Victoria were selected based on its functional annotation (**Supplementary Table 1**) and its expression during reproductive stages in *A. thaliana* (Klepikova et al., 2016; Mergner et al., 2020) and in *E. curvula* (Garbus et al., 2017). Thus, genes related to ploidy, expressed during floral development and genes previously described as involved in apomixis were used as templates for primers design. For this task, IDT Primer Quest online tool (https://www.idtdna.com/Primerquest/Home/Index) was used.

## 3 Results

### 3.1 Genome size determinations

**Figure 2:**
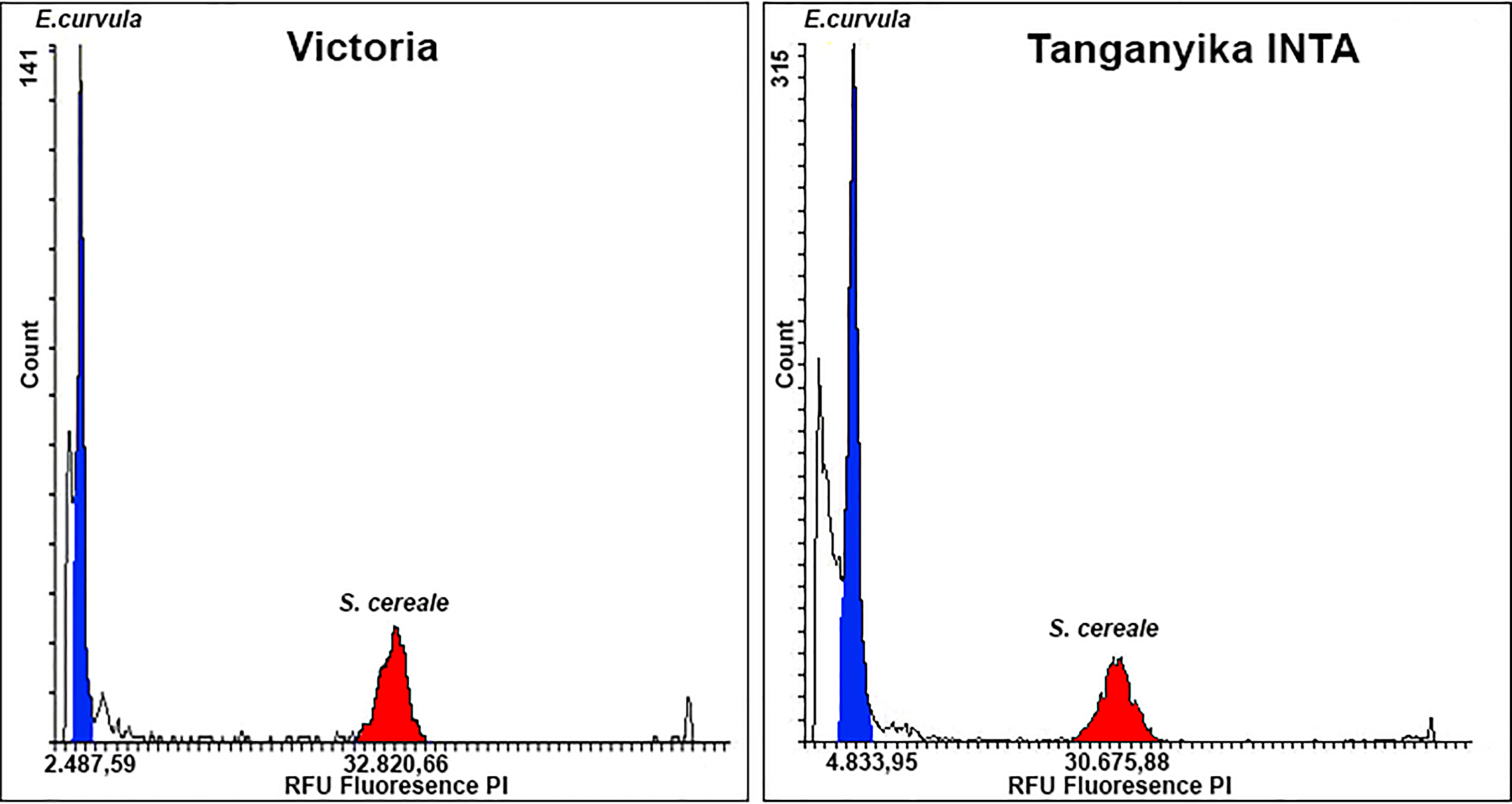
Flow cytometry histogram to assess *E. curvula* genome size. Y-axis shows nuclei count, while Y-axis display propidium iodide (PI) fluorescence. *E. curvula* and *S. cereale* are highlighted in blue and red, respectively. Victoria genotype presented half of the Tanganyika INTA RFU obtaining a genome length of 604.3 and 1,247.5 Mb, respectively.

As was expected, Victoria (2n=2x=20) showed half of the Tanganyika INTA (2n=4x=40) genome size (**Figure 2**). In terms of fluorescence, Victoria and Tanganyika displayed 2,487.59 and 4,833.95 RFU, respectively. The genome size in base pairs, estimated by using the *S. cereale* reference standard (7,917 Mb), was on average 604.3 (CV=6.33) and 1,247.5 (CV=6.7) Mb for the haploid genome for Victoria and Tanganyika INTA genomes, respectively.

### 3.2 Pangenome assembly and annotation

**Figure 3:**
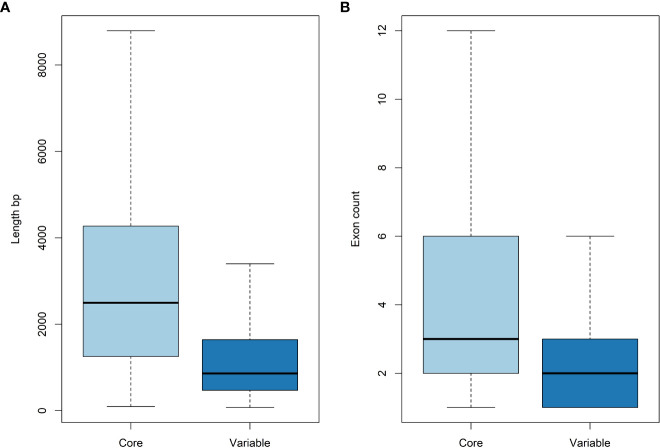
Gene length **(A)** and number of exons **(B)** of core and variable genes. Variable genes are shorter and have less exons than core genes.

Tanganyika INTA whole genome sequencing rendered 266,042,616 2x250 bp Illumina reads (~118 Gbp). These reads were mapped against the Victoria diploid genome assembly using Bowtie2 (**Figure 1**) (Langmead and Salzberg, 2012). A total of 24,473,069 (9.2%) reads did not map and were *de novo* assembled with Masurca software (Zimin et al., 2013). This assembly resulted in 18,102 contigs, which were filtrated based on contaminations and identities (see *Materials and Methods*) in 18,032 contigs. The total length of the assembly was 28,982,419 bp, and that of the N50 was 2,176 bp. The annotation rendered 3,952 gene models with an N50 of 1,158 bp. These genes are considered the variable genes, since they are not present in Victoria. This accounts for ~7% of the total annotated genes (56,469) from the Victoria genome assembly. As in other studied species (Golicz et al., 2016b; Ruperao et al., 2021), the variable genes are significatively shorter and have less exons than the core genes based on t-tests (**Figure 3**).

**Figure 4:**
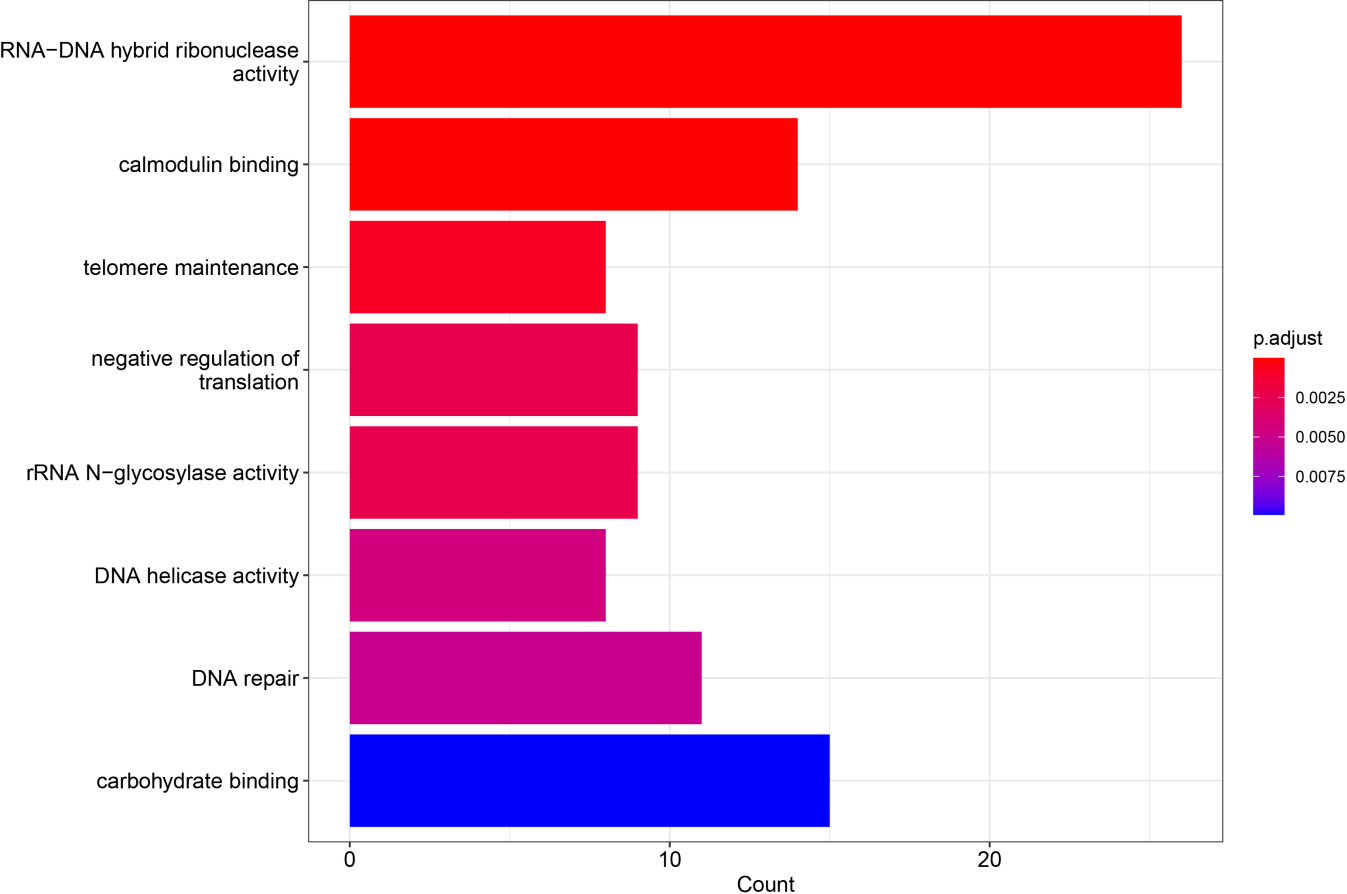
Differentially enriched GO terms of *E. curvula* pangenome variable genes. p-value cutoff was set in 0.05. The GO terms in the were sorted based on p-value. Three terms related to reproduction were found enriched.

In order to detect terms related to ploidy or reproduction, GO enrichment analysis was performed over the 3,952 variable genes (**Figure 4**) (**Supplementary Table 2**). Interestingly, the differentially enriched terms included those genes related to cell division such as DNA repair, telomere maintenance, and DNA helicase activity, suggesting that within the lost genes, genes associated with reproductive pathways are included.

### 3.3 Gene presence/absence

**Figure 5:**
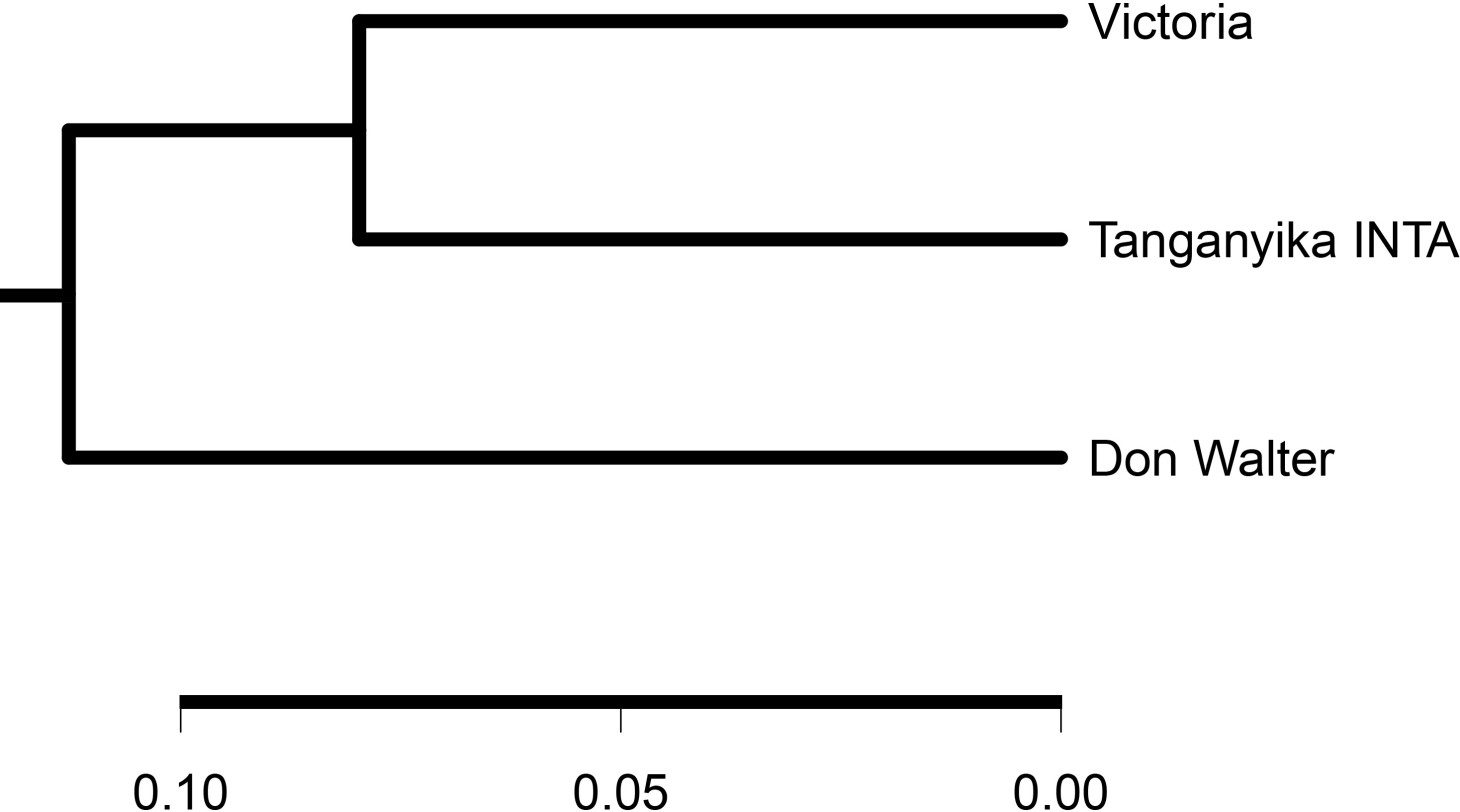
Phylogenetic tree of *Eragrostis curvula* including Victoria, Tanganyika INTA, and Don Walter INTA cvs. As expected, Tanganyika INTA is closer than Don Walter genotype.

The gene presence/absence variations (PAVs) analysis was made with SGSGeneLoss software (Golicz et al., 2015), mapping Tanganyika INTA reads against the pangenome. In theory, all Victoria’s genes should be present in Tanganyika INTA, since it originated from this genotype. Nevertheless, 958 gene models (1.64%) of the Victoria genes were not found in Tanganyika INTA. Out of this, only 67 gene models aligned against the UniProt database and were not enriched in any pathway (**Supplementary Table 3**). PAVs were also assessed using transcriptomic reads from Don Walter cv., which is apomictic, finding that 53,208 genes of the pangenome present in Victoria, Tanganyika INTA, and Don Walter genotypes (core genes). A phylogenetic tree was constructed with these data being Tanganyika INTA and Victoria closer than Don Walter (**Figure 5**).

### 3.4 Variant calling

**Figure 6:**
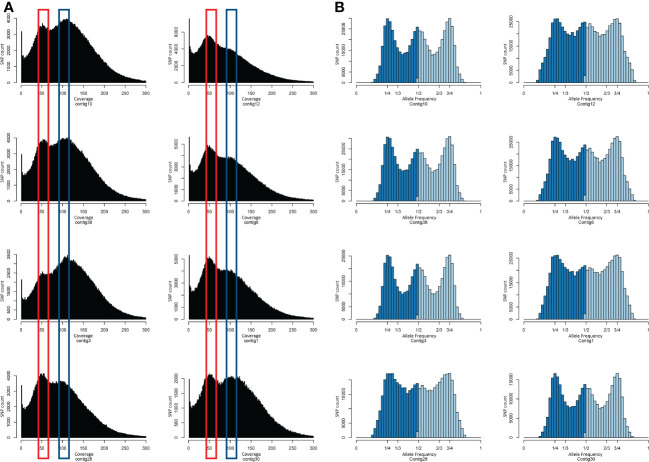
Tanganyika INTA SNP coverage **(A)** and allele frequency distribution **(B)** on the eight longest chromosomes of the Victoria genome assembly. **(A)** The y-axis represents the number of SNP and the x-axis the SNP coverage. The red and blue shapes highlight the two peaks around 50× and 100× showing the SNPs with diploid and tetraploid coverage. **(B)** The y-axis represents the number of SNP and the x-axis the allele frequency.

The variant calling was performed by mapping Tanganyika INTA reads against the reference genome assembly. After filtering, a total of 6,679,446 SNPs were called using GATK software (McKenna et al., 2010). The coverage of the SNPs and the allele frequency were especially analyzed in order to understand the representation of the tetraploid genome onto the diploid one. Interestingly, the coverage of Tanganyika’s SNPs over the Victoria chromosomes displayed two peaks, representing the diploid and haploid coverage (**Figure 6A**). However, the coverage was not equally distributed in all the chromosomes, with contig12, contig6, contig1, contig28, and contig30 being predominantly haploid and contig10, contig38, and contig3 diploid. The allele frequency shows a peak mainly at one-fourth and three-fourths of the distribution, even though in contigs with increased haploid coverage, the frequency of one-half was significant. From these data, it seems that Tanganyika INTA has both allotetraploid and autotetraploid behavior. Another phenomenon affecting these plots is that a certain degree of heterozygosity is expected between alleles, so it was not possible to distinguish between sub-genome differences and heterozygosity.

### 3.5 Variable gene validation

**Figure 7:**
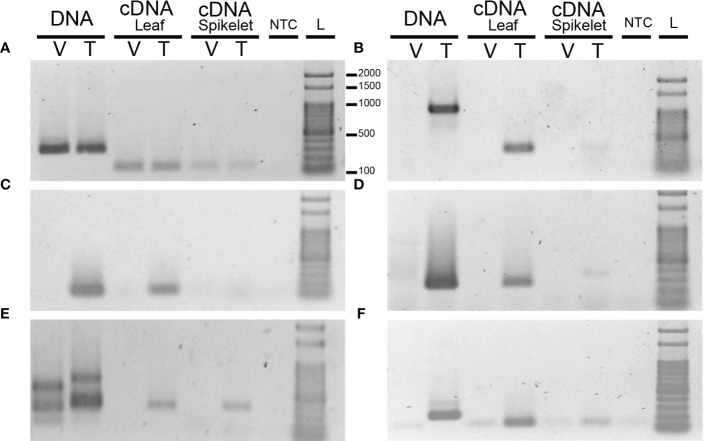
PCR amplification using DNA and cDNA from leaves and spikelets of *E*. *curvula*, Victoria (V), and Tanganyika (T) cvs. UBICE (ubiquitin conjugating enzyme transcript) **(A)** was used as positive control, and primers were designed over the annotated genes **(B)** *ABD1*, **(C)** *PVA12*, **(D)** *MEE66*, **(E)** *ATXR2*, and **(F)** *RECQ4A*. NTC (negative control) and L (Ladder). Presence/absence of the five genes was validated.

The variable gene models were functionally annotated using Uniprot database. In this way, five genes associated with reproduction and ploidy were selected for validation (**Supplementary Table 1**). Thus, PCR analysis was conducted using cDNA from leaves and spikelets and gDNA from spikelets extracted from Tanganyika INTA and Victoria genotypes (**Figure 7**).

Degradation *via* ubiquitination is a pathway associated with reproduction (Okada et al., 2013; Zühl et al., 2019; Selva et al., 2020). In this way, the gene *ABD1* (*ABA-hypersensitive DCAF1*, *AT4G38480*), which serves as a CUL4 E3 ligase substrate receptor, is only present in Tanganyika INTA and expressed in both leaves and spikelets (**Figure 7B**). Even though during the functional characterization of *ABD1*, the reproductive development was not assessed (Seo et al., 2014), this gene is involved in the regulation of abscisic acid (ABA), which was previously associated with embryo sac development (Chen et al., 2020). Therefore, the absence of *ABD1* could change the concentration of ABA during embryo sac development, altering the reproductive performance.

The gene *PVA12* was also not found in Victoria (**Figure 7C**). This gene interacts specifically with DC1 domain-containing protein, which produce vacuoleless gametophytes (D'ippólito et al., 2017). The lack of the vacuole in both male and female gametophytes produce arrest before the first round of mitosis after meiosis. As in Victoria, normal gametophyte development was found when *PVA12* was mutated, suggesting that other genes could have the same function.

Maternal effect embryo (MEE) arrest are genes required for embryo development and embryogenesis. *MEE66* was found in gDNA and cDNA of Tanganyika INTA and absent in Victoria (**Figure 7D**). The mutation of this gene in *A. thaliana* produces arrest at the two-cell embryo stage (Pagnussat et al., 2005).

Another gene not present in Victoria was *ATXR2*. This gene was only amplified in cDNA, since the primers were specifically designed on the splice junction (**Figure 7E**). *ATXR2* repress *de novo* shoot organogenesis activating the expression of *ARR5* and *ARR7 via* H3K36me3 marks in their promoters (Lee et al., 2021). When *ATXR2* is mutated, *ARR5* and *ARR7* are not expressed and shoot regeneration is improved, since these genes activate *WUS* expression. Even more, during embryogenesis, *ARR* genes are also activated (Müller and Sheen, 2008), suggesting that the lack of *ATXR2* in Victoria could distort the normal embryo development.

A key gene related to reproduction and probably with ploidy was *RECQ4A*, a DNA helicase that repairs double-stranded breaks during DNA replication. Although faint bands, product of dimers, were found in gDNA and cDNA of Victoria, the expected amplicon size for RECQ4A was only observed in Tanganyika INTA cDNA and gDNA (**Figure 7F**). In meiosis, double-stranded breaks are catalyzed by *SPO22-1* and *SPO11-2* genes, which are part of the MiMe complex used to produce mitosis instead of meiosis in rice and *A. thaliana* (d'Erfurth et al., 2009; Mieulet et al., 2016). Loss of function of *RECQ4A* in *A. thaliana* produces chromatin bridges between non-homologous chromosomes producing defective pollen and reduced fertility, whereas non-alterations during recombination were observed (Higgins et al., 2011). Even though it was not very frequent, *recq4a* mutant showed fragments of chromosomes due to the tension of the chromatin bridges between non-homologous chromosomes.

## 4 Discussion

In this study, a pangenomic approach was adopted to unravel genome diversity in *E. curvula* species. Even though this grass is studied as an apomictic model (Carballo et al., 2021b), here, we focused on the alterations produced by a recent diploidization event (Echenique et al., 1996) of the tetraploid cv. Tanganyika INTA to produce the Victoria genotype, obtained by *in vitro* culture of inflorescences (Cardone et al., 2006). The ploidy reduction brought about the change in reproductive mode from facultative apomictic in Tanganyika INTA to sexual in the diploid Victoria.

Using flow cytometry, the genome size of both the diploid Victoria and the tetraploid Tanganyika INTA genotypes were measured, resulting in ~600 and ~1,250 Mb, respectively. These values were expected for tetraploid and diploid *E. curvula* genotypes and were in concordance with previous measurements (Zappacosta et al., 2019). Interestingly, the genome size of Tanganyika INTA was bigger than that of the allotetraploid *E. tef* (~800 Mb) and closer to that of *E. nindensis* (1,035 Mb) (Hundera et al., 2000; Pardo et al., 2020).

As in other species, a mapping approach was used to assemble the new contigs (Golicz et al., 2016b; Gao et al., 2019; Ruperao et al., 2021). The mapping rate of Tanganyika INTA was close to 92%, and the new pangenome increased the length up to 28,982,419 bp. The newly annotated gene models classified as variable were 3,952. The SGSGeneLoss software also found that only 958 of the gene models (1.64%) present in Victoria were not found in Tanganyika INTA. Only 64 out of 958 genes found a match against UniProt database and were not particularly enriched in any term or pathway. Since Victoria originated from Tanganyika INTA, in theory, all the genes present in the tetraploid genome should be present in the diploid. However, it is known that some biases are produced during the sequencing, especially in polyploid genomes, that could produce loss of coverage in certain genomic regions (Schatz et al., 2012). Even more, as in most of the genome assemblies, small contaminations that are hard to detect could be inserted in the Victoria genome assembly responsible for the genes absent in Tanganyika INTA (Steinegger and Salzberg, 2020).

There are two main forces responsible for natural diploidization in plant species: cytological diploidization and genic diploidization (Ma and Gustafson, 2005). Cytological diploidization produces chromosomal rearrangements that eventually produce homologous pairing and exclude homologous pairing. On the other hand, genic diploidization is the consequence of gene deletions during evolution that lead to failures in chromosome pairing (Li et al., 2021b). Syntenic analysis between Victoria and *E. tef* showed that, although there are some rearrangements between genomes, most of the chromosomes are well conserved (Carballo et al., 2019). Given these results, it is logical to think that Victoria was produced by the regeneration of a haploid microspore. It is also important to note that even though Tanganyika INTA female gametophyte has approximately 95% of apomeiosis, the male meiosis is completely normal (Rodrigo et al., 2017). Therefore, probably, Victoria was regenerated from a male gamete that, after recombination, lost the genes responsible for the apomictic development. Even more, the sexual pathway seems to be affected as well, since Victoria has decreased fertility and yield. The external genetic and epigenetic changes applied by the tissue culture could also be responsible for the variations in the reproductive pathway (Madlung and Comai, 2004; Bednarek and Orłowska, 2020). A plausible explanation for the decrease in the reproductive performance in Victoria is that the sexual reproduction could not overcome the loos of the apomictic pathway.

The SNP coverage over the chromosome length contigs in Victoria showed a binomial distribution with one peak around 50× (representing haploid coverage) and other close to 100×. This supports the idea that Tanganyika INTA is a segmental allotetraploid with a sub-genome coverage peak at 50×, and both alleles are covered at the 100× peak (**Figure 6A**). Interestingly, not all the chromosomes show the same distribution; Contig12, Contig6, Contig1, Contig30, and Contig28 are predominantly covered by one sub-genome, whereas Contig10, Contig38, and Contig3 are fully covered. This is in agreement with the statement that allopolyploidy and autopolyploidy show continuous variation in sub-genome divergence rather than being independent processes (Stebbins, 1947; Madlung and Comai, 2004). In fact, in many different species, it was observed that both auto- and allopolyploids present certain degree of multi- and bivalent pairing during meiosis (Li et al., 2021b). Allele frequency correlates with the genome coverage, showing diploid-like trend in contigs with predominantly 50× of coverage and tetraploid like distribution in the 100× covered contigs (**Figure 6B**). A similar behavior was shown in *Spartina pectinate*, where genotypes classified as allotetraploid displayed a polysomic inheritance (Crawford et al., 2016). The results obtained in this work suggest that Tanganyika INTA is segmental allotetraploid, whereas in other *E. curvula* cultivars, they are classified as both allotetraploid and segmental allotetraploid, based on cytological observations of the meiosis pairing (Poverene, 1988).

Out of the 3,952 gene models, *ABD1*, *PVA12*, *MEE66*, *ATXR2*, and *RECQ4A* were selected to be used as templates for PCR primer design. These genes were validated in both gDNA and cDNA from leaves and spikelets from Tanganyika INTA and Victoria. Even though the presence/absence of *ATXR2* in cDNA is clear, several non-specific bands were amplified in gDNA of Victoria and Tanganyika INTA, suggesting that these primers also amplify in repetitive regions that are hard to sequence and assembly not present in *E. curvula* databases. All the genes assessed here are, at some point, associated with both reproduction and ploidy level. Regarding the genes that are absent in Victoria, it is important to note that after diploidization, the reproductive performance of Victoria was seriously affected (Carballo et al., 2021b). The lack of some genes such as *RECQ4A*, *MEE66*, and *PVA12*, which are directly involved in the reproductive pathway, could be the cause of the reduction in fertility. Particularly, *RECQ4A* is a helicase, and its mutation in *A. thaliana* produces defective pollen and reduces fertility (Higgins et al., 2011). Moreover, *RECQ4A* mutants showed chromatin bridges between non-homologous chromosomes and enhanced homologous recombination in somatic cells (Hartung et al., 2007; Higgins et al., 2011).

In conclusion, the pangenome approach used here provides evidence about the *E. curvula* ploidy nature and complements cytological observations in previous works (Cardone et al., 2006), indicating that Tanganyika INTA is segmental allotetraploid. In addition, it gives information about the genes related to both reproduction and ploidy. The loose of the pathways involved in apomixis in Victoria could be associated with the disruption or lack of the genetic region linked to apomixis and/or the presence of epigenetic marks that are characteristics of a certain ploidy level. Moreover, the loose of genes involved in apomixis reduce the mechanisms available for reproduction, decreasing the fertility of Victoria.

## Data availability statement

The datasets presented in this study can be found in online repositories. The names of the repository/repositories and accession number(s) can be found below: https://www.ncbi.nlm.nih.gov/, BioProject: PRJNA914706, SRA: SRR22846089.

## Author contributions

Conception: JC, VE, DZ, and JS. Experimental design: JC, VE, and JS. Materials: JC, JS, and DZ. Experimental procedures: JC, AB, JS, and DZ. Bioinformatic data processing: JC and AG. Analysis and interpretation: JC, VE, MC, and DZ. Writing: JC, VE, MC, and DZ. Critical review: JC, EA, MC, and VE. All authors contributed to the article and approved the submitted version.
